# Supplementary material for: Ligand−Structure Effects on N−Heterocyclic Carbene Rhenium Photo− and Electrocatalysts of CO2 Reduction
Source: Molecules. 2023 May 17;28(10):4149. doi: 10.3390/molecules28104149 (PMC10221375; doi:10.3390/molecules28104149)
Supplement: Supplementary file 1 [file molecules-28-04149-s001.zip › molecules-2349487-supplementary.pdf]

Supporting Information for

Ligand-structure Effects on *N*-Heterocyclic Carbene Rhenium Photo- and Electrocatalysts of CO_2 Reduction

Lauren Kearney [1], Michael P. Brandon[1], Andrew Coleman[1], Ann M. Chippindale[2], František Hartl[2]*, Ralte Lalrempuia[1,3], Martin Pižl[4], Mary T. Pryce [1]*

Table of Contents

Structural Characterisation Data ……………..……………………………………………..2-6

Extended UV-Vis Absorption spectra, Excitation spectra and 77 K Emission spectra……….…………………………………………………………………..…....7-11

Lifetime Measurements……………………………………………………………...11-12

Additional Photophysical Characterisation of Re-NHC-1 and Re-NHC-2 ……………………………………………………………..……………………...12-13

Cyclic Voltammograms………………………….…….…………………………….14-15

DFT-Calculated IR spectra of Re-NHC-1, singly reduced radical anion and anionic secondary product…………………………………………………………………………………..15

Photocatalytic CO_2 Reduction…………………… ………………………………………16

^{1}H NMR and ^{13}C NMR Spectra ……………………….…………………………16-23

Table S1. Crystal data and structure refinement for Re-NHC-1 (see Chart 1 and Figure 1 in the main text).

Identification code	Re-NHC-1	
Molecular formula	$C_{23}H_{13}N_3O_3BrRe$	
Formula weight	645.48	
Temperature	100(1) K	
Wavelength	0.71073 Å	
Crystal system	Triclinic	
Space group	*P*–1 (#2)	
Unit cell dimensions	*a* = 9.13630(1) Å	α = 65.357(3)°
	b = 10.98913(2) Å	β = 82.916(2)°
	c = 11.34750(2) Å	γ = 82.988(2)°
Volume	1024.46(2) $Å^3$	
Z	2	
Density (calculated)	2.092 mg m^{-3}	
Absorption coefficient	7.912 mm^{-1}	
Crystal habit	Yellow block	
Crystal size	0.015 × 0.026 × 0.044 mm	
Theta range for data collection	2.0452 to 30.7435°	
Index ranges	–13<=*h*<=13, –15<=*k*<=14, –15<=*l*<=14	
Reflections collected	23208	
Independent reflections	5320 [*R*(int) = 0.040]	
Completeness to θ = 25.210°	100.0 %	
Absorption correction	Multi-scan	
Max. and min. transmission	0.888 and 0.781	

Refinement method	Full-matrix least-squares on *F*
Data / restraints / parameters	4395 / 1 / 283
Goodness-of-fit on F^2	1.107
Final *R* indices [$I > 3\sigma(I)$]	$R1$ = 0.0230, $wR2$ = 0.0216
Largest diff. peak and hole	0.93 and –0.62 $eÅ^{-3}$
CCDC cif deposition number	CCDC 2244937

Table S2. Selected bond lengths (Å) and angles (°) for complex Re-NHC-1.

Re(1) – Br(2)	2.6457(4)	Br(2) – Re(1) – N(1)	82.61(7)
Re(1) – N(1)	2.196(2)	Br(2) – Re(1) – C(3)	177.28(9)
Re(1) – C(3)	1.940(3)	N(1) – Re(1) – C(3)	95.49(11)
Re(1) – C(5)	1.974(4)	Br(2) – Re(1) – C(5)	91.10(10)
Re(1) – C(7)	1.915(3)	N(1) – Re(1) – C(5)	96.58(12)
Re(1) – C(21)	2.117(3)	C(3) – Re(1) – C(5)	91.04(13)
O(4) – C(3)	1.097(4)	Br(2) – Re(1) – C(7)	94.12(10)
O(6) – C(5)	1.140(4)	N(1) – Re(1) – C(7)	172.88(12)
O(8) – C(7)	1.149(4)	C(3) – Re(1) – C(7)	87.55(13)
N(1) – C(26)	1.339(4)	C(5) – Re(1) – C(7)	89.79(13)
N(1) – C(30)	1.341(4)	Br(2) – Re(1) – C(21)	85.83(8)
N(9) – C(10)	1.407(4)	N(1) – Re(1) – C(21)	73.92(10)
N(9) – C(21)	1.387(4)	C(3) – Re(1) – C(21)	91.79(12)
N(9) – C(26)	1.419(4)	C(5) – Re(1) – C(21)	170.31(12)
N(20) – C(19)	1.392(4)	C(7) – Re(1) – C(21)	99.59(12)
N(20) – C(21)	1.340(4)	Re(1) – N(1) – C(26)	117.1(2)
C(10) – C(11)	1.444(4)	Re(1) – N(1) – C(30)	123.5(2)
C(10) – C(19)	1.373(4)	Re(1) – C(21) – N(9)	116.5(2)
C(11) – C(12)	1.416(4)	Re(1) – C(21) – N(20)	138.9(2)
C(11) – C(16)	1.412(5)	Re(1) – C(3) – O(4)	178.7(3)
C(12) – C(13)	1.374(5)	Re(1) – C(5) – O(6)	178.8(3)
C(13) – C(14)	1.391(5)	Re(1) – C(7) – O(8)	177.4(3)
C(14) – C(15)	1.378(5)	C(26) – N(1) – C(30)	118.1(3)
C(15) – C(16)	1.417(5)	C(10) – N(9) – C(21)	110.6(2)
C(16) – C(17)	1.476(5)	C(10) – N(9) – C(26)	131.8(3)
C(17) – C(18)	1.415(4)	C(21) – N(9) – C(26)	115.5(3)

C(17) – C(25)	1.414(5)	C(19) – N(20) – C(21)	112.0(2)
C(18) – C(19)	1.419(4)	N(9) – C(10) – C(11)	133.2(3)
C(18) – C(22)	1.408(4)	N(9) – C(10) – C(19)	105.5(3)
C(22) – C(23)	1.364(5)	C(11) – C(10) – C(19)	120.8(3)
C(23) – C(24)	1.402(5)	C(10) – C(11) – C(12)	124.0(3)
C(24) – C(25)	1.363(5)	C(10) – C(11) – C(16)	116.4(3)
C(26) – C(27)	1.395(4)	C(12) – C(11) – C(16)	119.4(3)
C(27) – C(28)	1.371(5)	C(11) – C(12) – C(13)	120.4(3)
C(28) – C(29)	1.383(5)	C(12) – C(13) – C(14)	120.3(3)
C(29) – C(30)	1.377(5)	C(13) – C(14) – C(15)	120.3(3)
		C(14) – C(15) – C(16)	121.0(4)
		C(11) – C(16) – C(15)	118.0(3)
		C(11) – C(16) – C(17)	121.4(3)
		C(15) – C(16) – C(17)	120.5(3)
		C(16) – C(17) – C(18)	119.9(3)
		C(16) – C(17) – C(25)	122.8(3)
		C(18) – C(17) – C(25)	117.3(3)
		C(17) – C(18) – C(19)	116.5(3)
		C(17) – C(18) – C(22)	120.2(3)
		C(19) – C(18) – C(22)	123.2(3)
		N(20) – C(19) – C(10)	107.0(3)
		N(20) – C(19) – C(18)	128.8(3)
		C(10) – C(19) – C(18)	124.2(3)
		N(9) – C(21) – N(20)	104.6(3)
		C(18) – C(22) – C(23)	120.6(3)
		C(22) – C(23) – C(24)	119.7(3)
		C(23) – C(24) – C(25)	120.6(3)
		C(17) – C(25) – C(24)	121.5(3)
		N(1) – C(26) – N(9)	114.1(3)
		N(1) – C(26) – C(27)	122.4(3)
		N(9) – C(26) – C(27)	123.1(3)
		C(26) – C(27) – C(28)	118.0(3)
		C(27) – C(28) – C(29)	120.3(3)
		C(28) – C(29) – C(30)	118.0(3)
		N(1) – C(30) – C(29)	123.1(3)

Table S3. Selected experimental and calculated (*in vacuo*) bond lengths (Å) for the complex Re-NHC-1.

Lengths	Experimental	Calculated
Re(1) – Br(2)	2.6457(4)	2.686
Re(1) – N(1)	2.196(2)	2.244
Re(1) – C(3)	1.940(3)	1.927
Re(1) – C(5)	1.974(4)	1.989
Re(1) – C(7)	1.915(3)	1.932
Re(1) – C(21)	2.117(3)	2.115
O(4) – C(3)	1.097(4)	1.167
O(6) – C(5)	1.140(4)	1.158
O(8) – C(7)	1.149(4)	1.164
N(1) – C(26)	1.339(4)	1.353
N(1) – C(30)	1.341(4)	1.348
N(9) – C(10)	1.407(4)	1.418
N(9) – C(21)	1.387(4)	1.387
N(9) – C(26)	1.419(4)	1.413
N(20) – C(19)	1.392(4)	1.391
N(20) – C(21)	1.340(4)	1.347
C(10) – C(19)	1.373(4)	1.379

Table S4. Selected experimental and calculated (*in vacuo*) bond angles (°) for complex Re-NHC-1.

Angles	Experimental	Calculated
Br(2) – Re(1) – N(1)	82.61(7)	82.5
Br(2) – Re(1) – C(3)	177.28(9)	177.4
N(1) – Re(1) – C(3)	95.49(11)	95.0
Br(2) – Re(1) – C(5)	91.10(10)	89.3
N(1) – Re(1) – C(5)	96.58(12)	97.8
C(3) – Re(1) – C(5)	91.04(13)	91.8
Br(2) – Re(1) – C(7)	94.12(10)	91.1
N(1) – Re(1) – C(7)	172.88(12)	168.6
C(3) – Re(1) – C(7)	87.55(13)	91.3
C(5) – Re(1) – C(7)	89.79(13)	91.5
Br(2) – Re(1) – C(21)	85.83(8)	85.7
N(1) – Re(1) – C(21)	73.92(10)	73.2
C(3) – Re(1) – C(21)	91.79(12)	92.9
C(5) – Re(1) – C(21)	170.31(12)	170.1
C(7) – Re(1) – C(21)	99.59(12)	97.0
Re(1) – N(1) – C(26)	117.1(2)	116.0
Re(1) – N(1) – C(30)	123.5(2)	124.0
Re(1) – C(21) – N(9)	116.5(2)	118.0
Re(1) – C(21) – N(20)	138.9(2)	137.2
Re(1) – C(3) – O(4)	178.7(3)	179.6
Re(1) – C(5) – O(6)	178.8(3)	178.2
Re(1) – C(7) – O(8)	177.4(3)	176.6
C(26) – N(1) – C(30)	118.1(3)	118.2
C(10) – N(9) – C(21)	110.6(2)	110.5
C(10) – N(9) – C(26)	131.8(3)	132.1
C(21) – N(9) – C(26)	115.5(3)	115.8
C(19) – N(20) – C(21)	112.0(2)	112.2
N(9) – C(10) – C(19)	105.5(3)	105.4
N(20) – C(19) – C(10)	107.0(3)	107.0
C(10) – C(19) – C(18)	124.2(3)	123.8
N(9) – C(21) – N(20)	104.6(3)	104.7
N(1) – C(26) – N(9)	114.1(3)	113.83

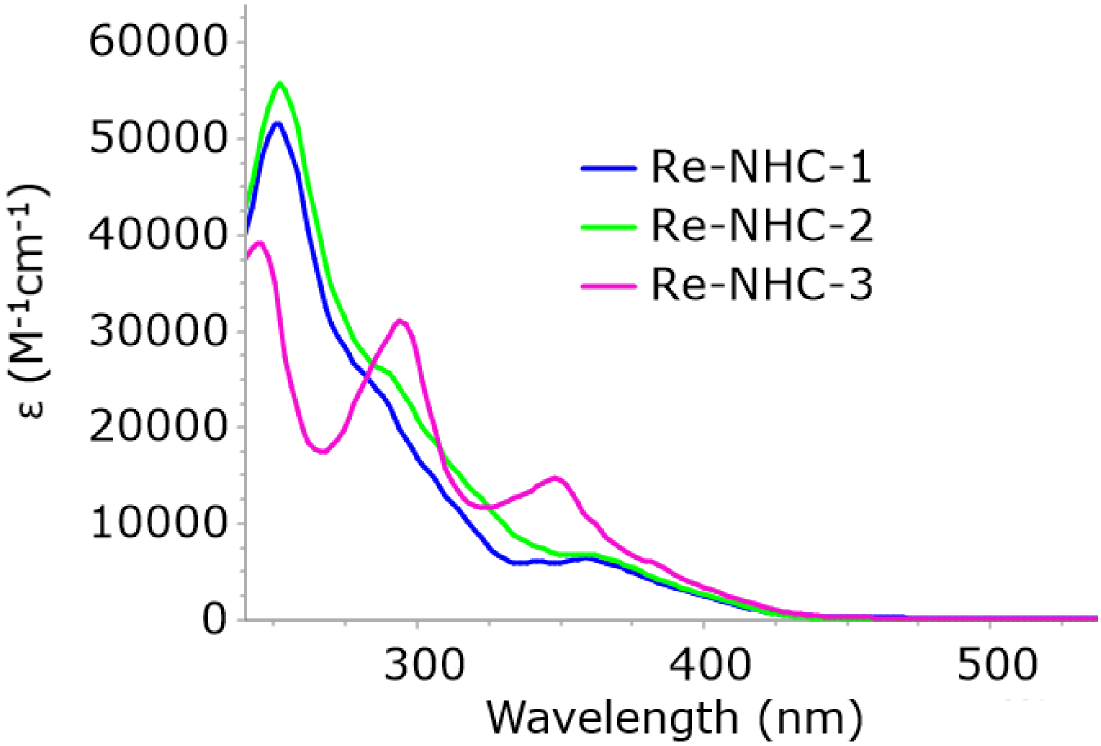

Figure S1. UV-Vis absorption spectra of Re-NHC−1−3 in dry acetonitrile at room temperature.

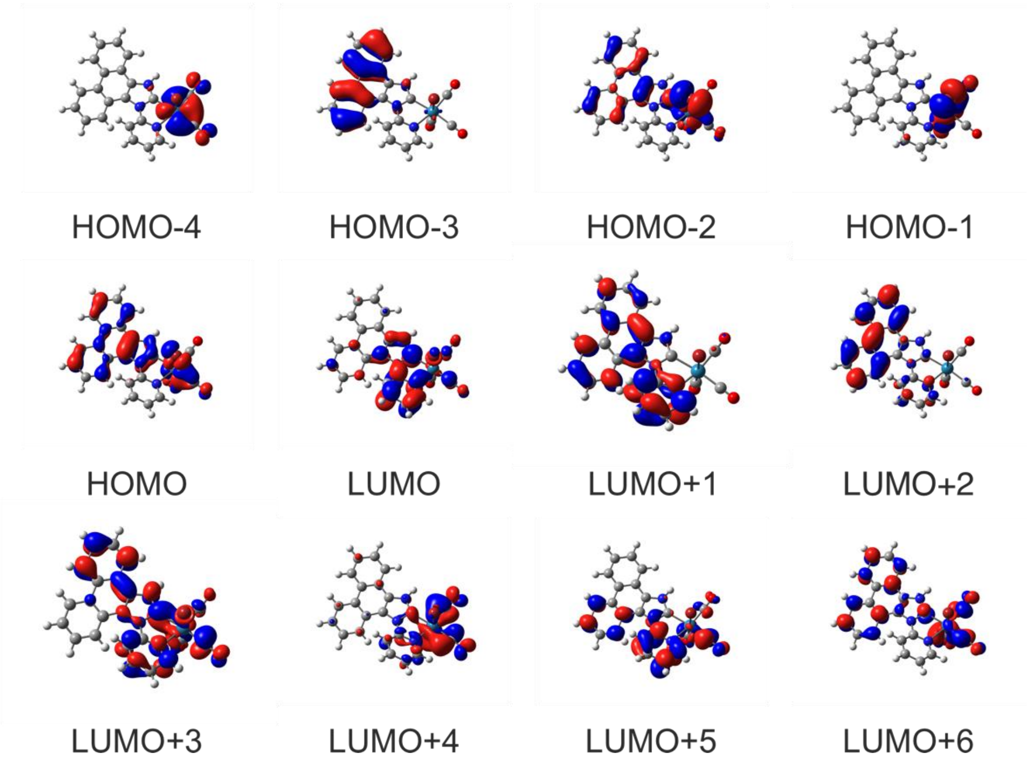

Figure S2. Molecular orbitals involved in electronic transitions detected in the experimental UV-Vis absorption spectrum of Re-NHC-1.

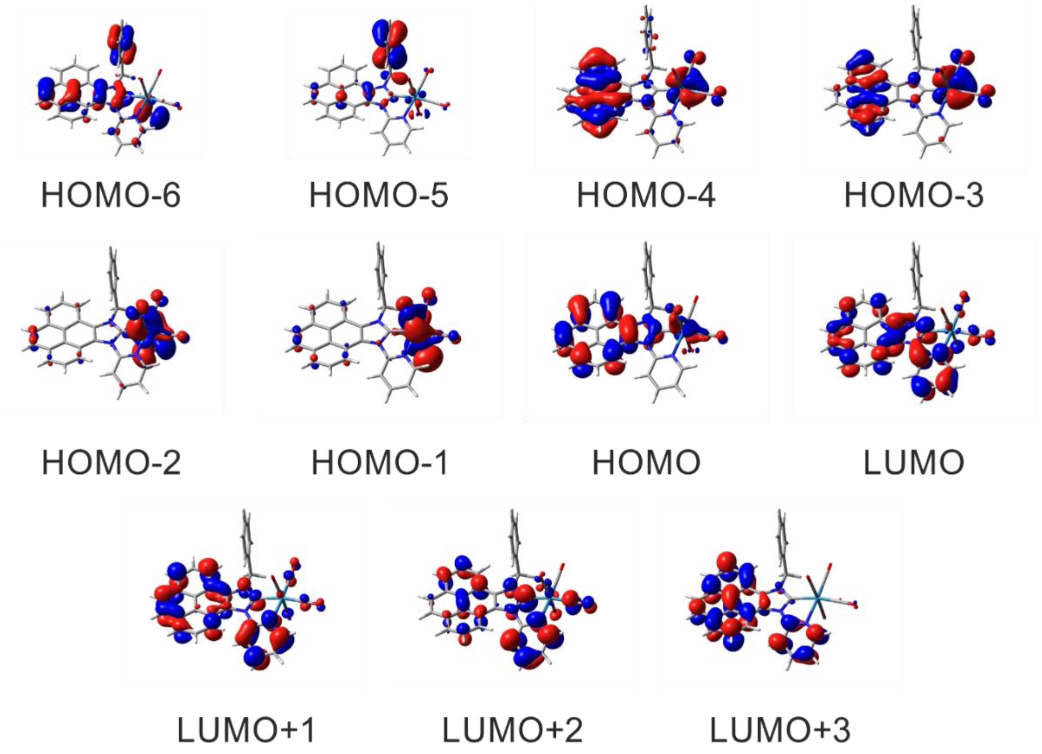

Figure S3. Molecular orbitals involved in electronic transitions detected in the experimental UV-Vis absorption spectrum of Re-NHC-3.

Table S5. Major electronic excitations in Re-NHC-1 determined by TD-DFT

Complex	Wavelength (nm)[a]	OS (f)[b]	Major contributions (%)
**Re-NHC-1**	402 (405sh)	0.0563	HOMO → LUMO (66)
	376 (358)	0.067	HOMO-1 → LUMO (67)
	320 (290)	0.096	HOMO → LUMO+3 (50)<br>HOMO → LUMO+4 (33)
	314 (290)	0.1061	HOMO-3 → LUMO (51)<br>HOMO-1 → LUMO+1 (25)<br>HOMO → LUMO+2 (30)
	304 (290)	0.1053	HOMO-1 → LUMO+3 (31)<br>HOMO → LUMO+3 (30)<br>HOMO → LUMO+4 (36)
	276 (250)	0.1592	HOMO-3 → LUMO+1 (25)<br>HOMO → LUMO+6 (27)
	269 (250)	0.3558	HOMO-3 → LUMO+2 (31)<br>HOMO → LUMO+6 (32)
	265 (250)	0.3132	HOMO-3 → LUMO+2 (39)<br>HOMO-3 → LUMO+3 (33)<br>HOMO → LUMO+5 (27)
	263 (250)	0.2147	HOMO-3 → LUMO+3 (43)
	262 (250)	0.105	HOMO-4 → LUMO+2 (40)
	260 (250)	0.1761	HOMO-4 → LUMO+2 (39)<br>HOMO-2 → LUMO+4 (37)

[a] Corresponding experimental absorption in brackets. [b] OS = Oscillator Strength.

Table S6. Major electronic excitations in Re-NHC-3 determined by TD-DFT.

Complex	Wavelength (nm)[a]	OS (*f*)[b]	Major contributions (%)
**Re-NHC-3**	415 (400sh)	0.0815	HOMO-1 → LUMO (21)<br>HOMO → LUMO (62)<br>HOMO → LUMO+1 (23)
	380 (384sh)	0.0606	HOMO-2 → LUMO (24)<br>HOMO-1 → LUMO (56)<br>HOMO-1 → LUMO+1 (22)
	365 (348)	0.1495	HOMO → LUMO+1 (61)<br>HOMO-2 → LUMO (26)
	328 (328sh)	0.1412	HOMO → LUMO+2 (48)<br>HOMO → LUMO+3 (37)
	311 (295)	0.0939	HOMO-4 → LUMO (25)<br>HOMO-2 → LUMO+2 (24)<br>HOMO-1 → LUMO+2 (40)<br>HOMO → LUMO+3 (22)
	309 (295)	0.3027	HOMO-4 → LUMO (39)<br>HOMO-3 → LUMO (25)<br>HOMO-1 → LUMO+2 (33)<br>HOMO → LUMO+3 (21)
	302 (280sh)	0.0791	HOMO-6 → LUMO (44)<br>HOMO-5 → LUMO (35)<br>HOMO-3 → LUMO+1 (20)

[a] Corresponding experimental absorption in brackets. [b] OS = Oscillator Strength.

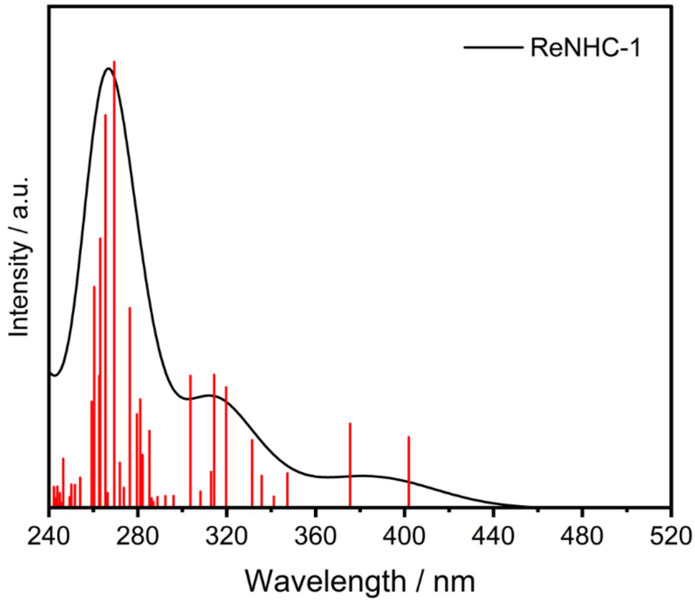

Figure S4. TD-DFT calculated electronic absorption spectrum of Re-NHC-1 in acetonitrile. The vertical excitations correspond to data in Table S5. The corresponding experimental spectrum in acetonitrile at 293 K is shown in Figure S1 (above) and Figure 2 (main text).

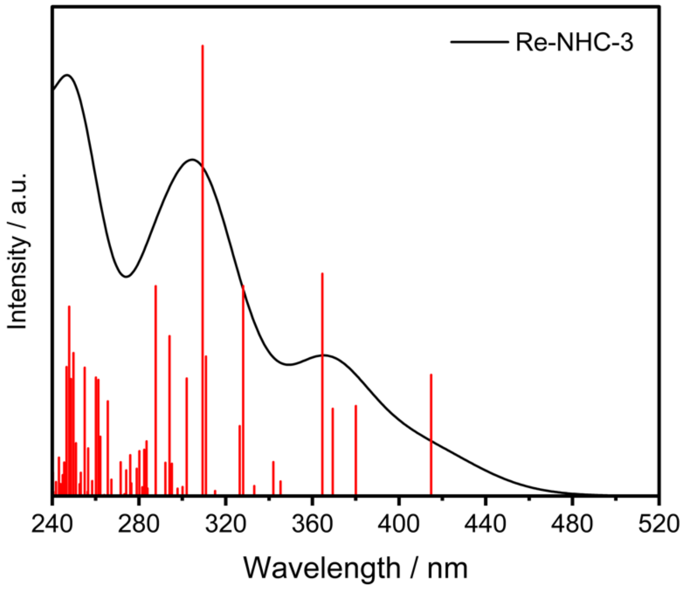

Figure S5. TD-DFT calculated electronic absorption spectrum of Re-NHC-3 in acetonitrile. The vertical excitations correspond to data in Table S6. The corresponding experimental spectrum in acetonitrile at 293 K is shown in Figure S1 (above) and Figure 2 (main text).

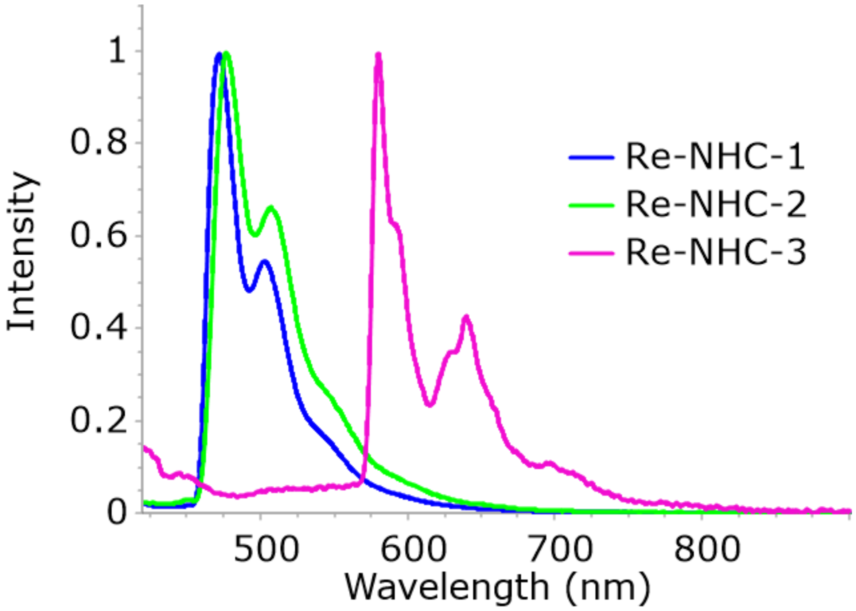

Figure S6. Emission spectra of **Re-NHC-1−3** in a beaker of ethanol−methanol 4:1 (v/v) at T = 77 K; λ_{exc} = 375 nm.

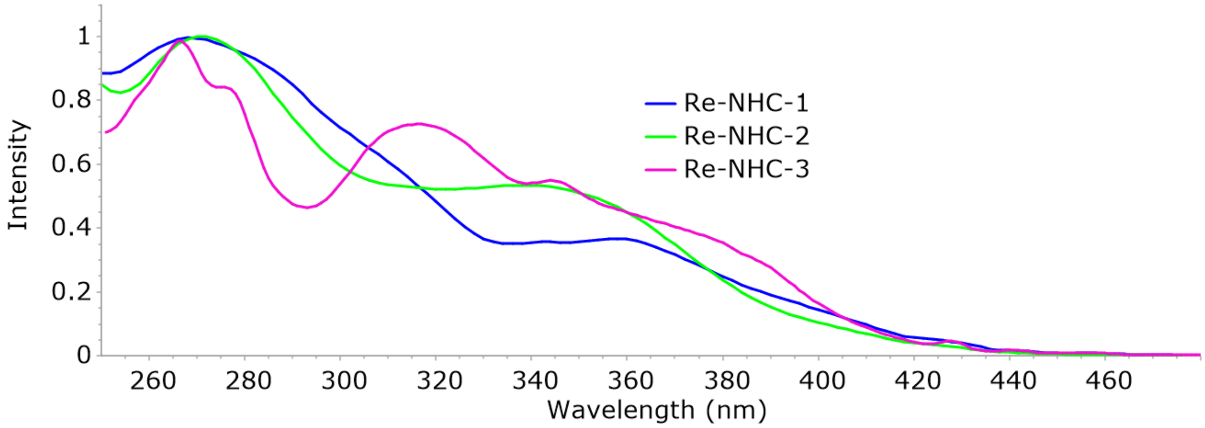

Figure S7. Excitation spectra of Re-NHC-1−3 in dry acetonitrile, λ_{em} = 490 nm.

All luminescence lifetime measurements (Figures S6−S8) were carried out in dry acetonitrile. The solutions were deaerated using the freeze-pump-thaw method. Excitation wavelength of 375 nm was used for all samples.

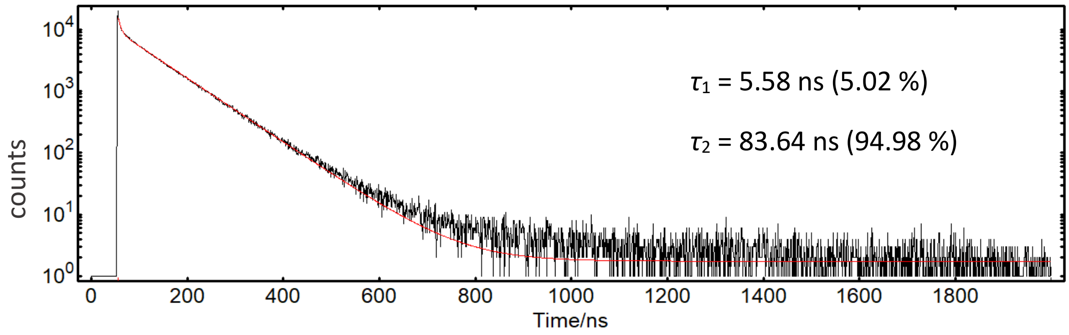

Figure S8. Emission decay of Re-NHC-1.

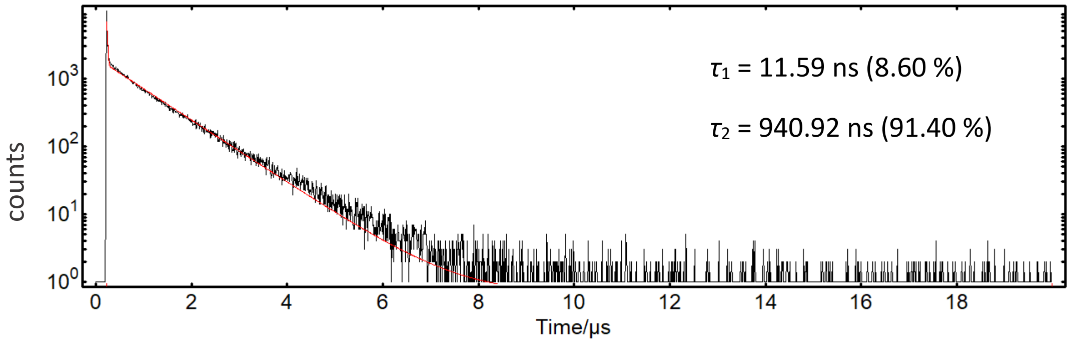

Figure S9. Emission decay of Re-NHC-2.

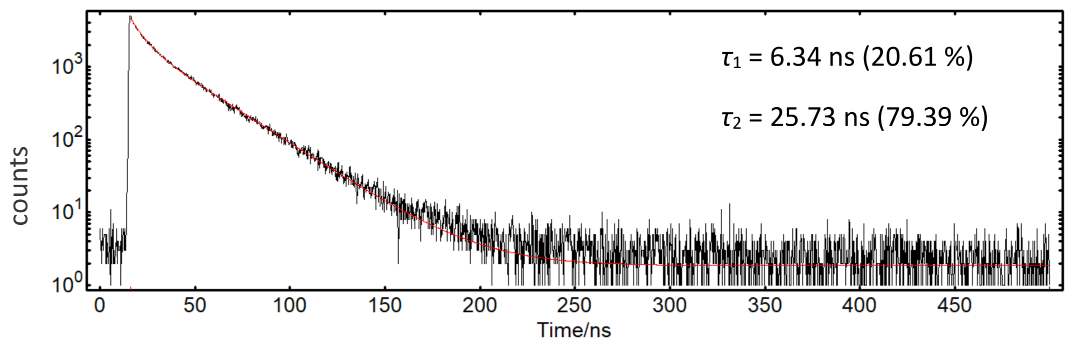

Figure S10. Emission decay of Re-NHC-3.

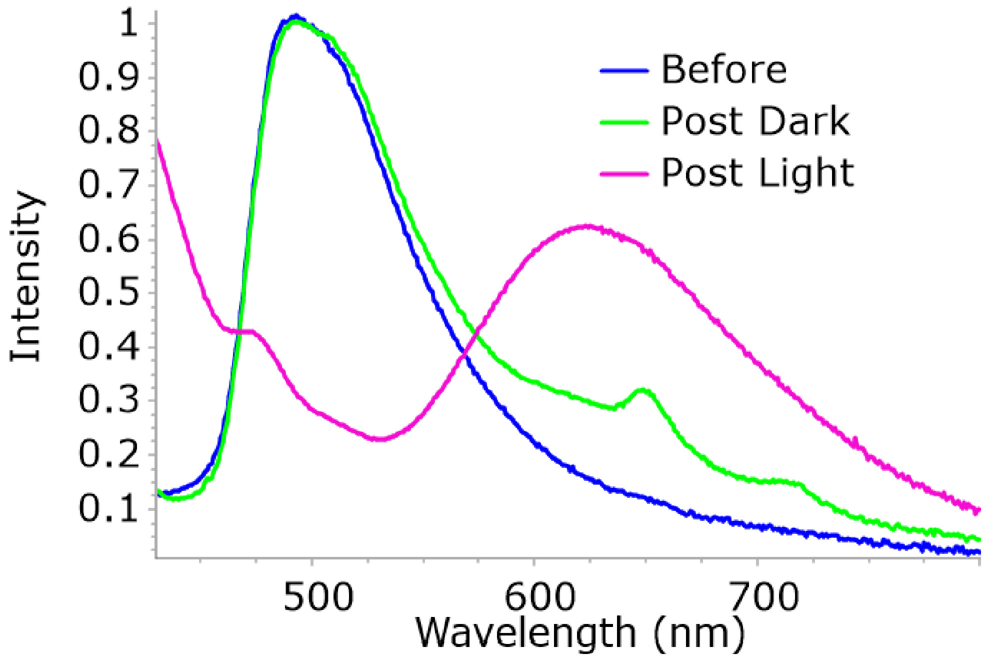

Figure S11. Changes observed in luminescence spectra of Re-NHC-1 in dry acetonitrile at ambient temperature (blue line) after standing 24 h in the dark (green line) and 24 h exposure to daylight/ceiling light (magenta line).

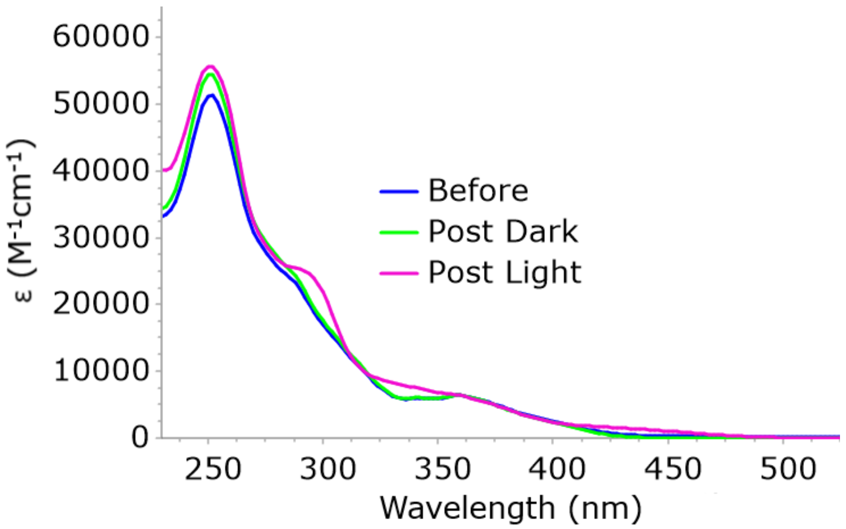

Figure S12. Changes observed in electronic absorption spectra of Re-NHC-1 in dry acetonitrile at ambient temperature (blue line) after standing 24 h in the dark (green line) and 24 h exposure to daylight/ceiling light (magenta line).

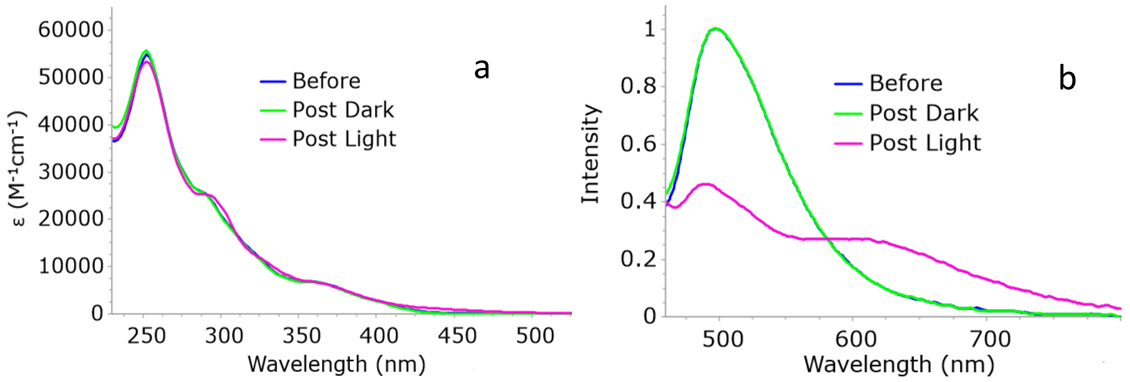

Figure S13. Changes in (a) electronic absorption, and (b) luminescence of Re-NHC-2 in dry acetonitrile at ambient temperature (blue lines) after standing 24 h in the dark (green lines) and 24 h exposure to daylight/ceiling light (magenta lines).

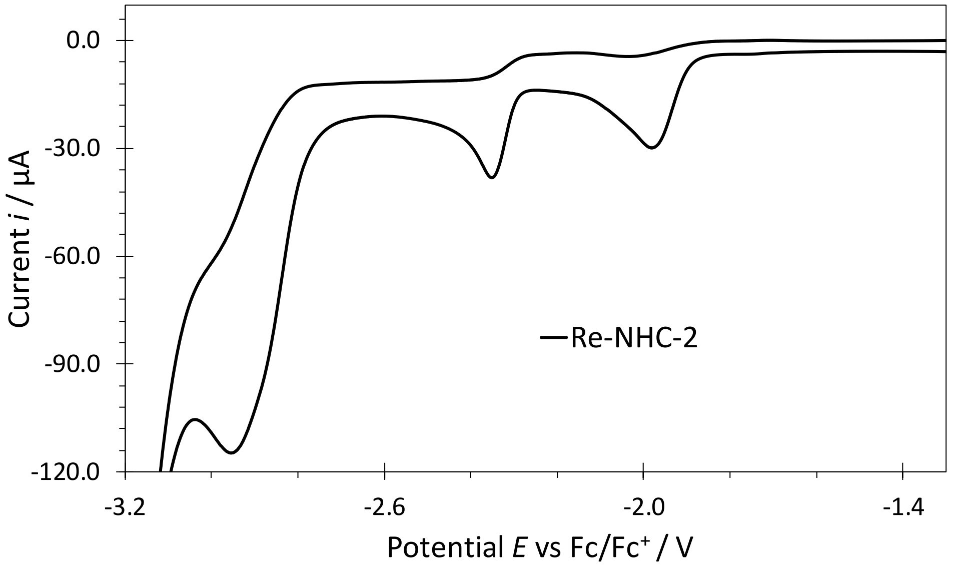

Figure S14. Cyclic voltammogram of 1 mM Re-NHC-2 in N_2-purged acetonitrile/10^{-1} M $TBAPF_6$, extended above −2.6 V (cf. Figure 4(a,c) in the main text). The scan rate was 100 mV s^{-1}.

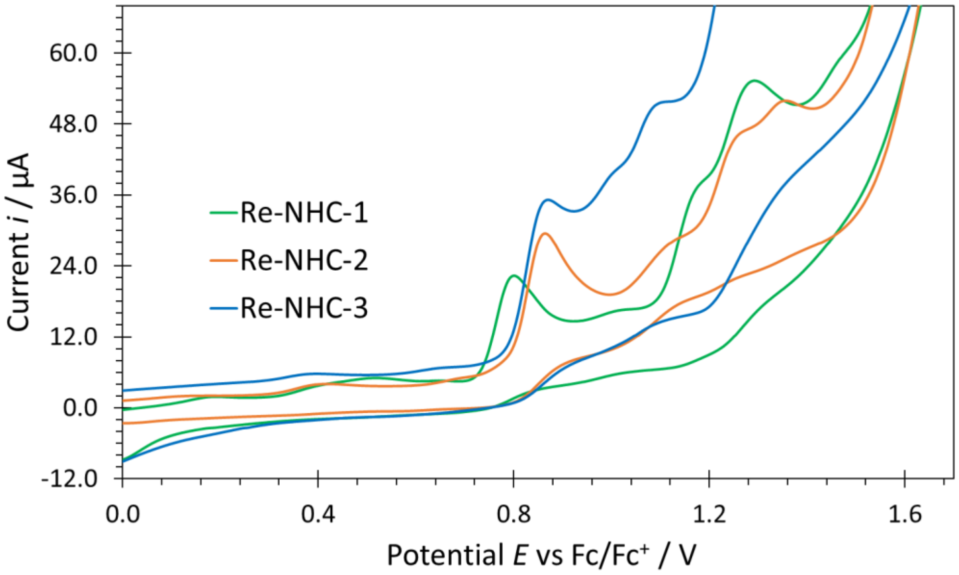

Figure S15. Cyclic voltammograms of 1 mM Re-NHC-1−3 (the oxidation potential range) in N_2-purged dry acetonitrile/10^{-1} M $TBAPF_6$. All measurements were performed at the scan rate of 100 mV s^{-1}.

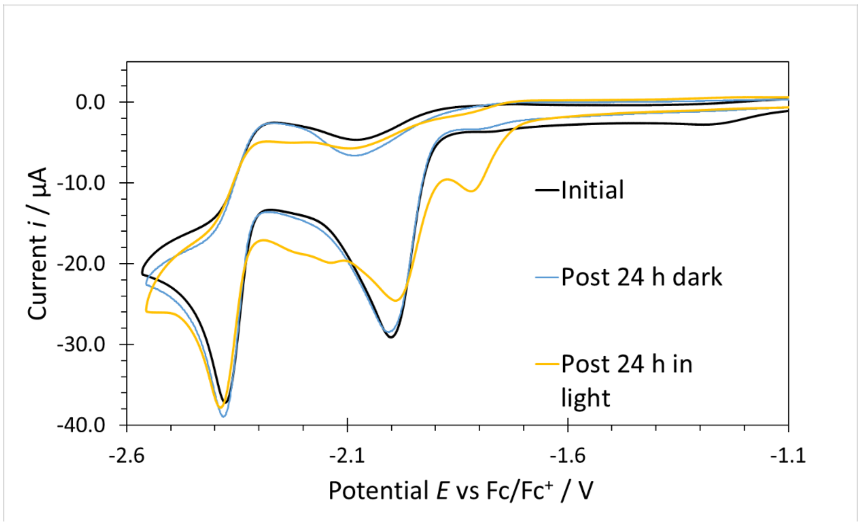

Figure S16. Changes in cyclic voltammograms of 1 mM Re-NHC-2 in N_2-purged dry acetonitrile/10^{-1} M $TBAPF_6$, recorded directly after electrolyte preparation (black line), after the storage in the dark for 24 h (blue line) and after the exposure to daylight for 24 h (yellow line). Scan rate of 100 mV s^{-1}.

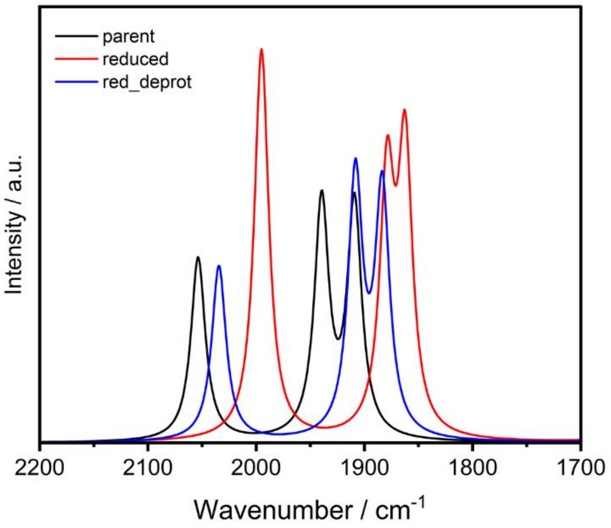

Figure S17. IR spectra in the CO-stretching region calculated by DFT for the parent complex Re-NHC-1 = [complex-Br] in the main text (black line) and the corresponding unstable singly reduced radical anion, $[\text{complex-Br}]^{\bullet-}$ (red line) converting instantaneously by reductive deprotonation (N−H bond cleavage) to the stable anionic secondary product, $[\text{complex}'\text{-Br}]^{-}$ (blue line).

Table S7: Photocatalytic conversion of CO_2 to CO using BIH as sacrificial electron donor after 24 hours of irradiation.

Catalyst	Solvent	λ_{irr} (nm)	TON
Re-NHC-1	DMF:MeOH (6:2, v/v)	355	11
Re-NHC-2	DMF:MeOH (6:2, v/v)	355	10
Re-NHC-3	DMF:MeOH (6:2, v/v)	470	26
Re-NHC-1	DMF:TEA (6:2, v/v)	355	0.14
Re-NHC-3	DMF:TEA (6:2, v/v)	355	1.64
Re-NHC-1	DMF:TEOA (6:2, v/v)	355	0.47
Re-NHC-3	DMF:TEOA (6:2, v/v)	355	6.22

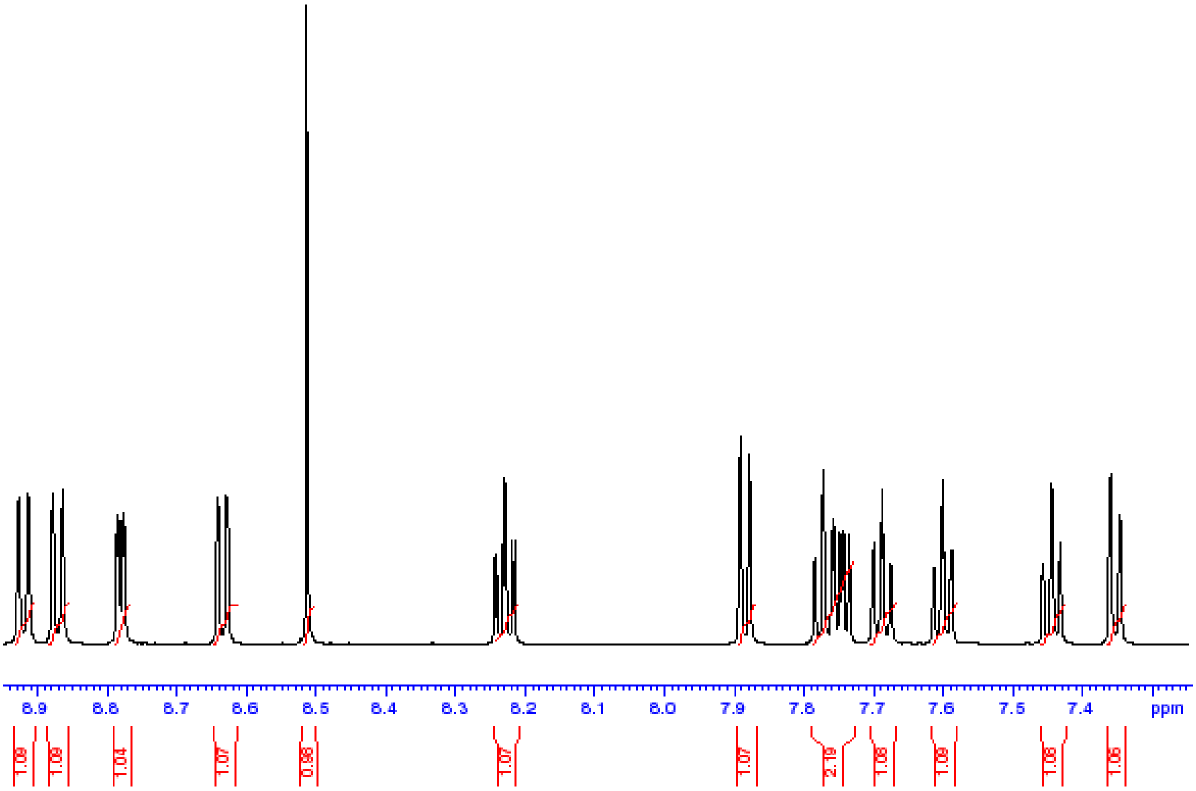

Figure S18. ^{1}H NMR spectrum of NHC-1 in DMSO-d_6, 600 MHz.

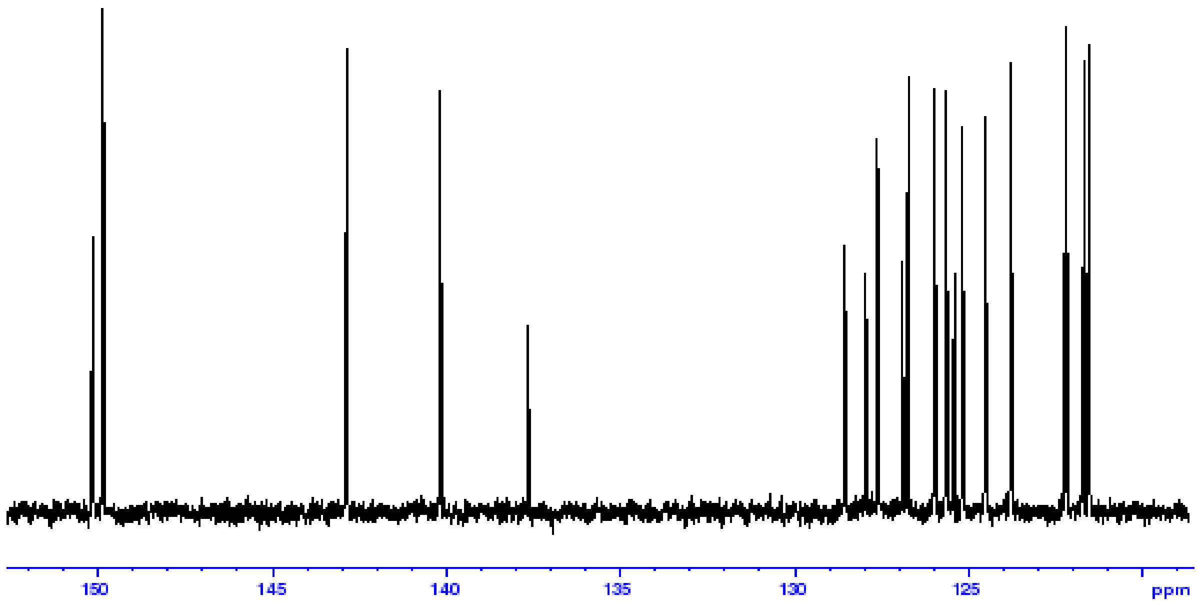

Figure S19. ^{13}C NMR spectrum of NHC-1 in DMSO-d_6, 150 MHz.

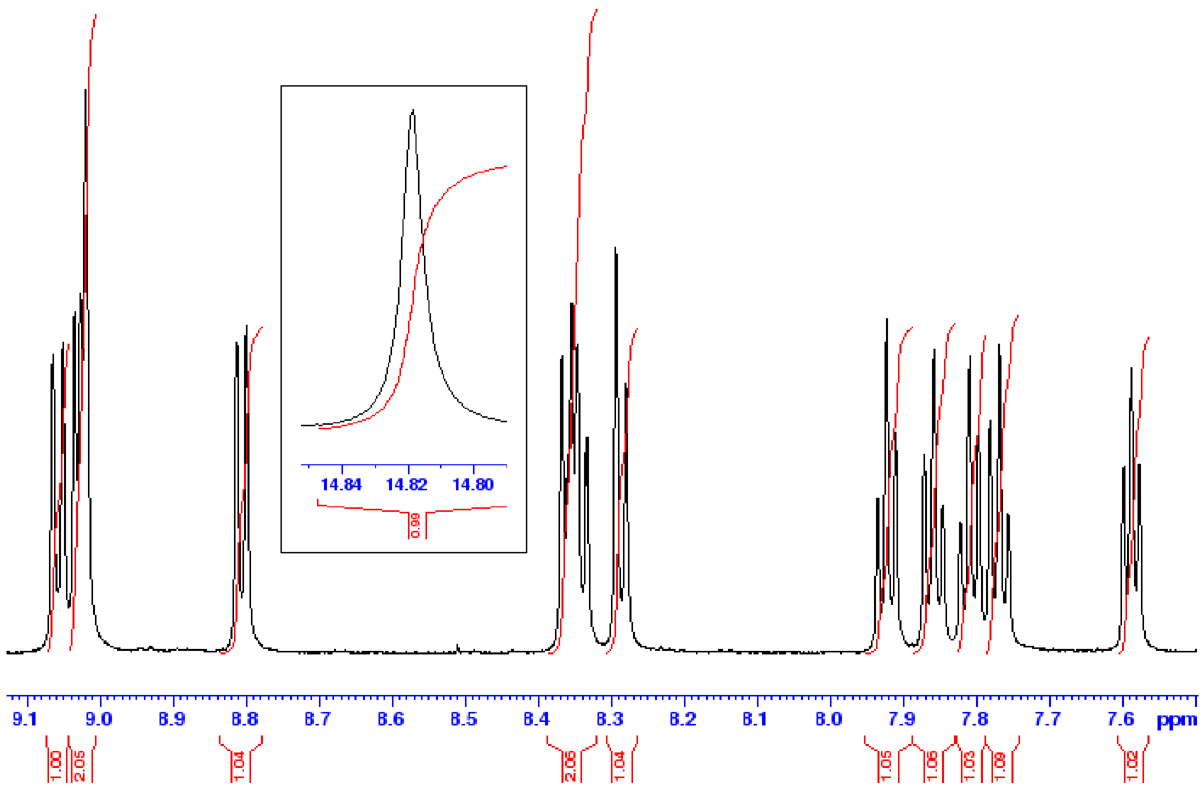

Figure S20. ^{1}H NMR spectrum of Re-NHC-1 in in DMSO-d_6, 600 MHz. The peak at ca. 14.8 ppm is included as an inset.

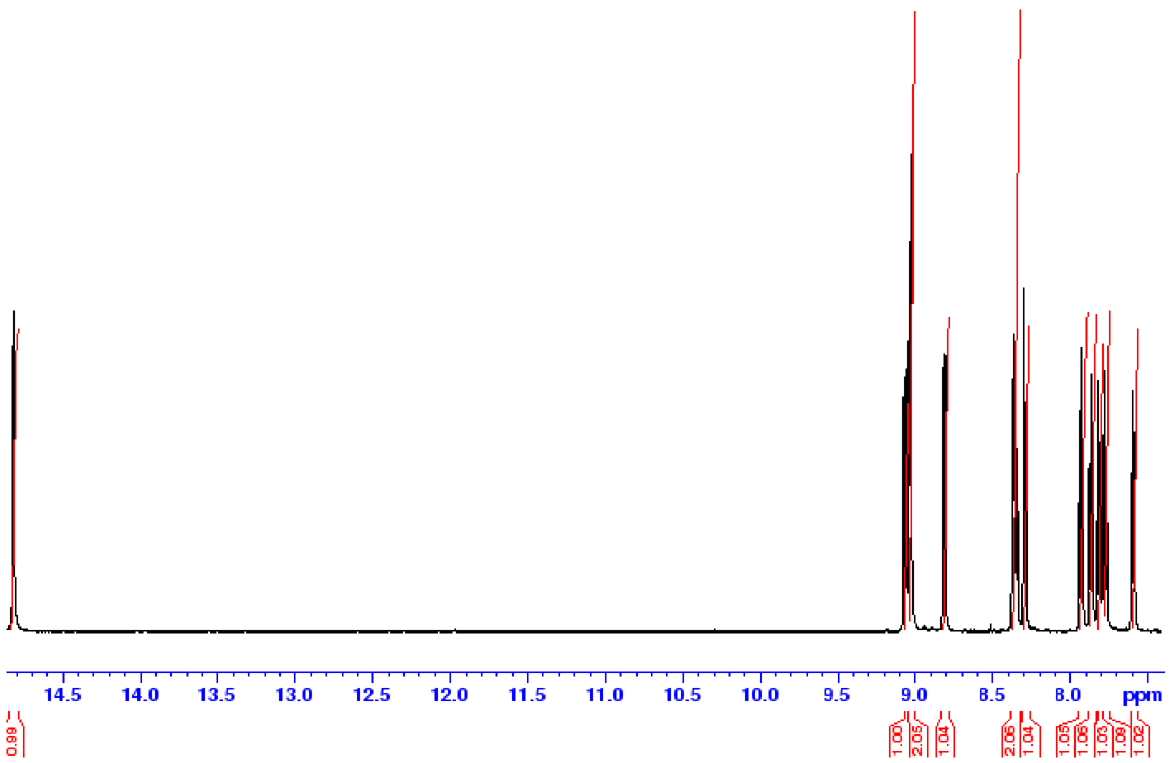

Figure S21. Extended ^{1}H NMR spectrum of Re-NHC-1 in DMSO-d_6, 600 MHz.

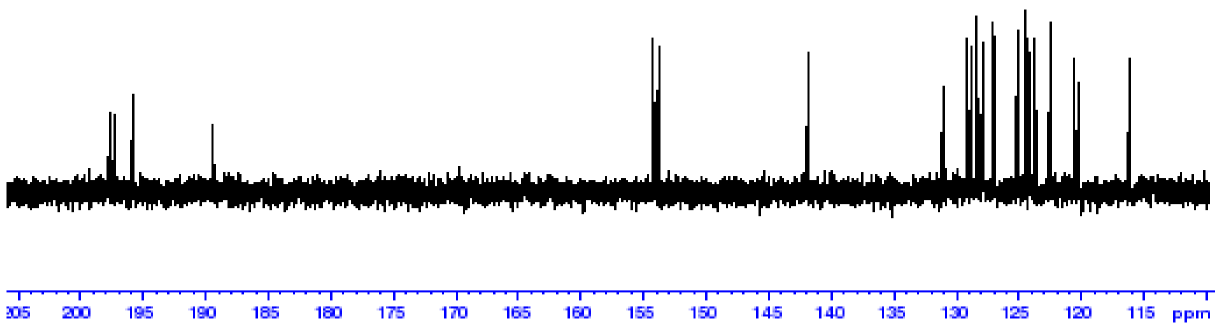

Figure S22. ^{13}C NMR spectrum of Re-NHC-1 in DMSO-d_6, 150 MHz.

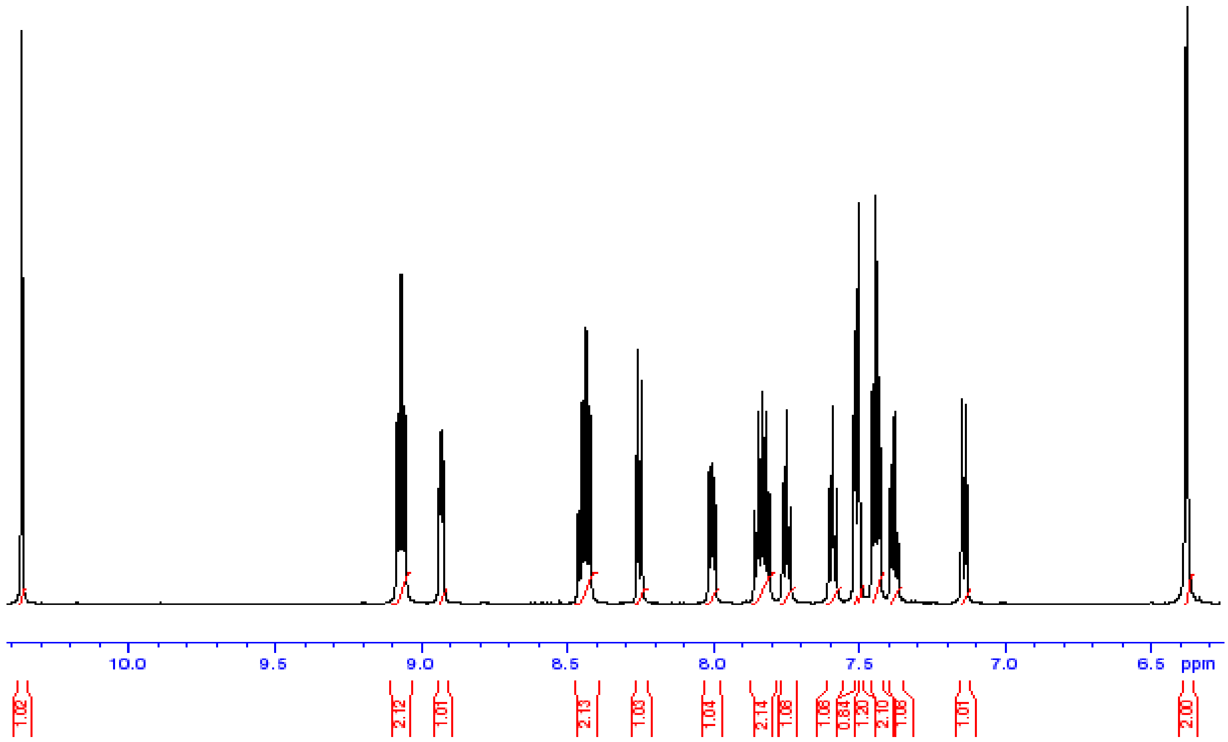

Figure S23. ^{1}H NMR spectrum of NHC-2 in DMSO-d_6, 600 MHz.

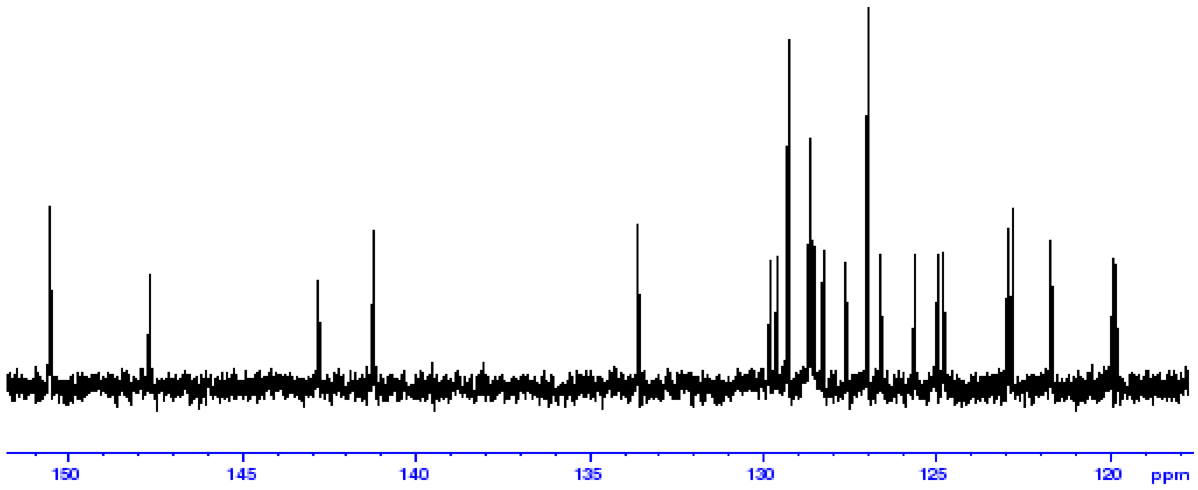

Figure S24. ^{13}C NMR spectrum of NHC-2 in DMSO-d_6, 150 MHz.

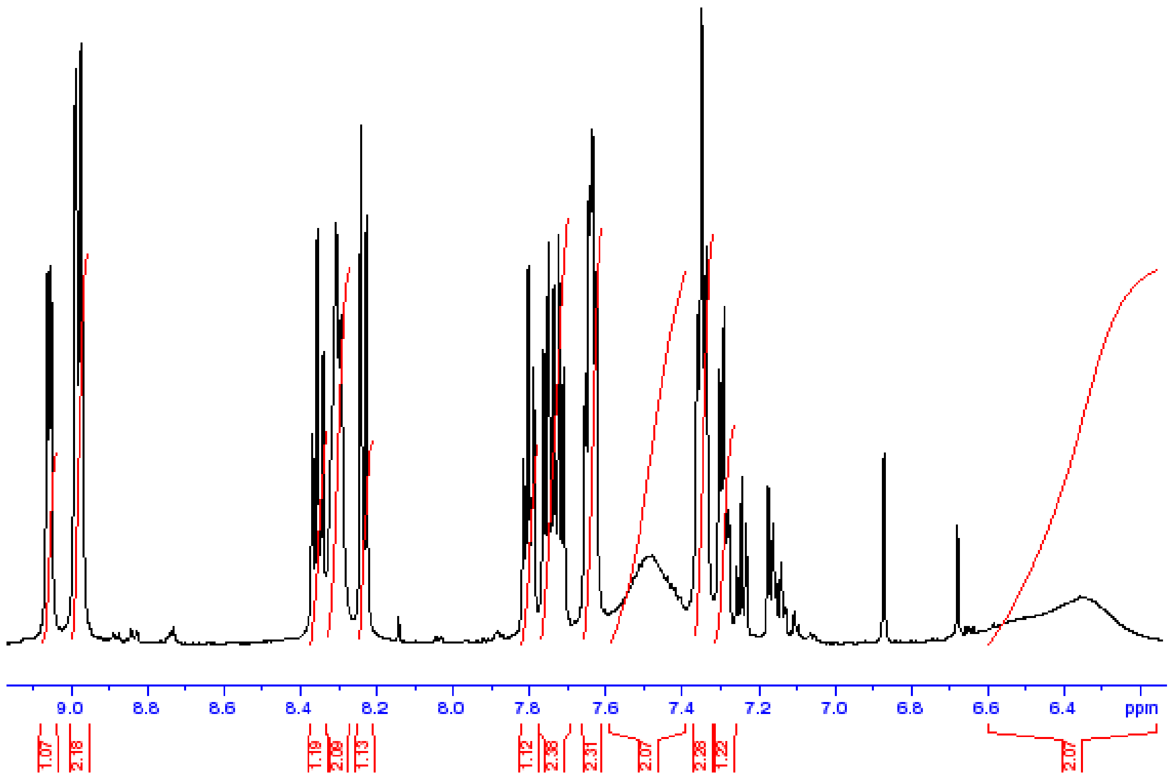

Figure S25: ^{1}H NMR spectrum of Re-NHC-2 in DMSO-d_6, 600 MHz.

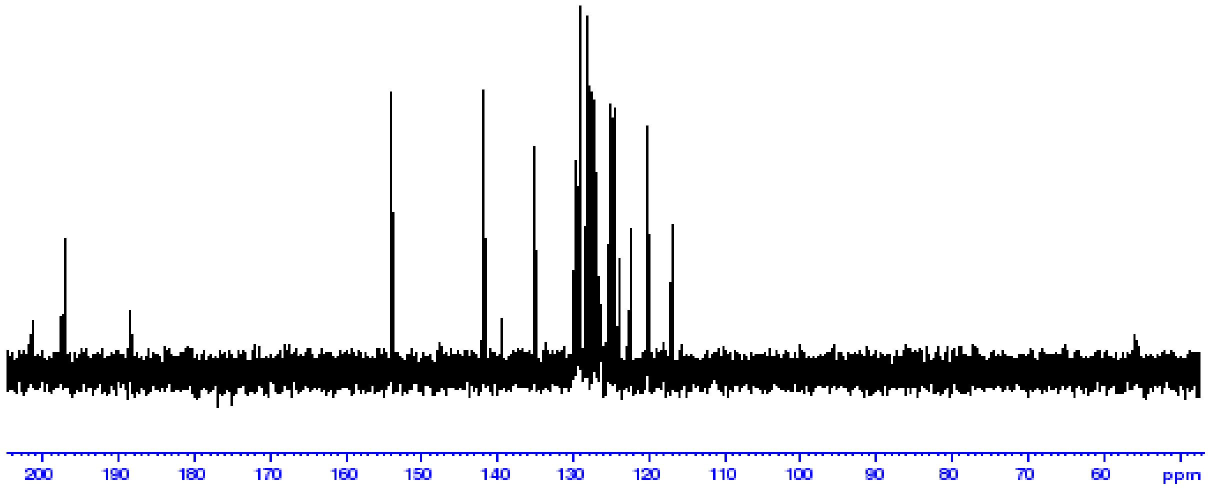

Figure S26. ^{13}C NMR spectrum of Re-NHC-2 in DMSO-d_6, 150 MHz.

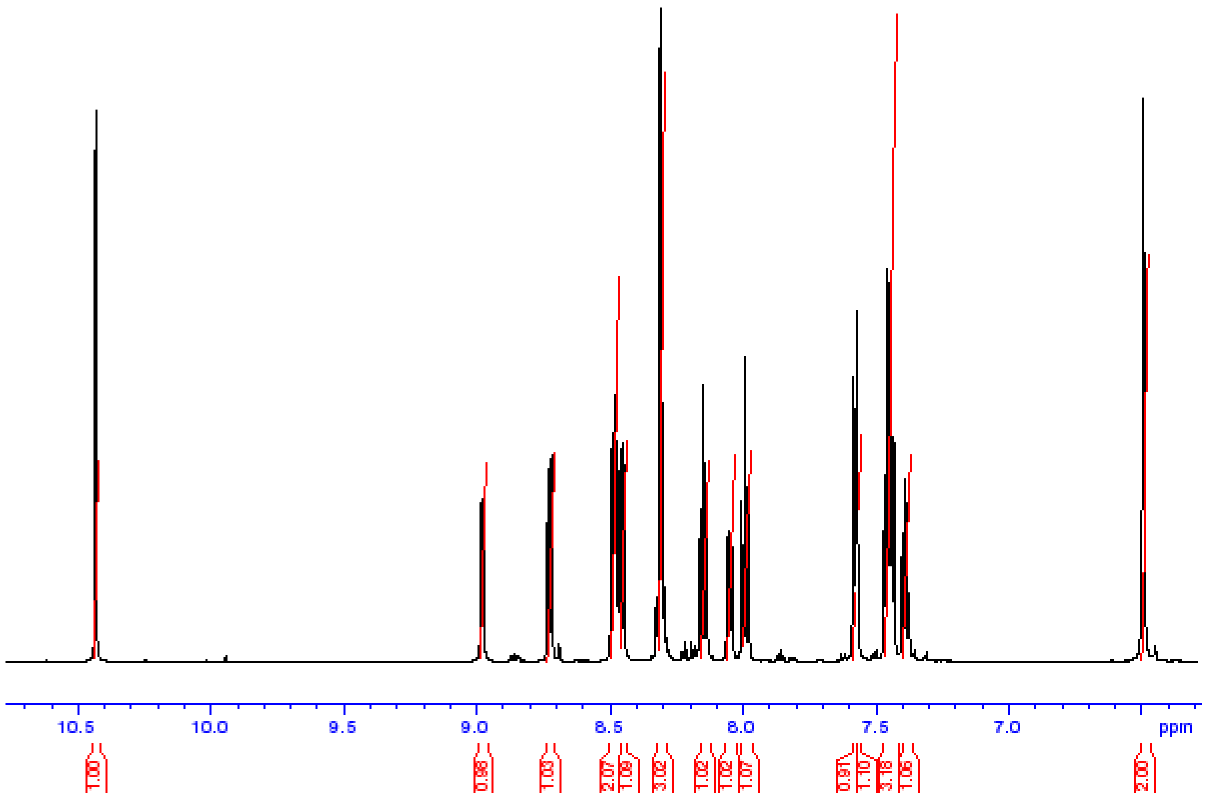

Figure S27. ^{1}H NMR spectrum of NHC-3 in DMSO-d_6, 600 MHz.

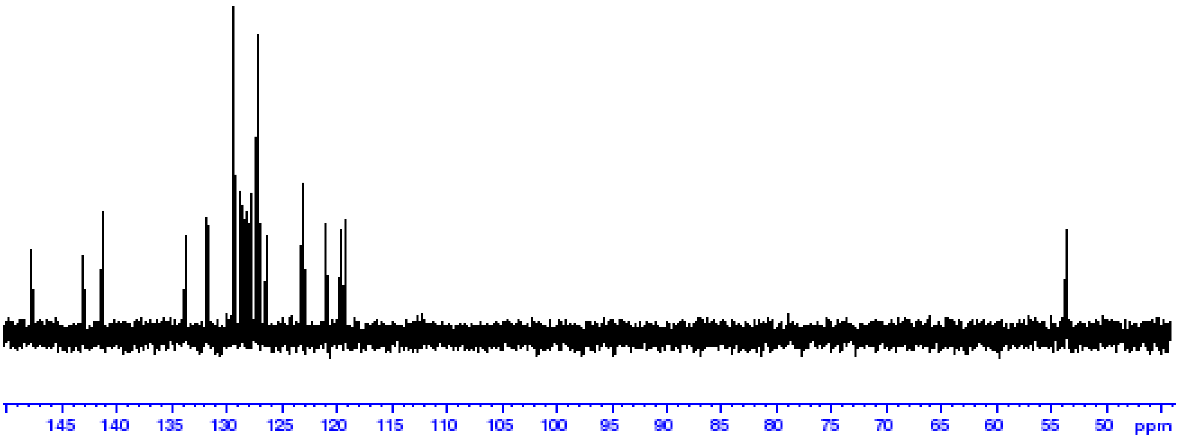

Figure S28. ^{13}C NMR spectrum of NHC-3 in DMSO-d_6, 150 MHz.

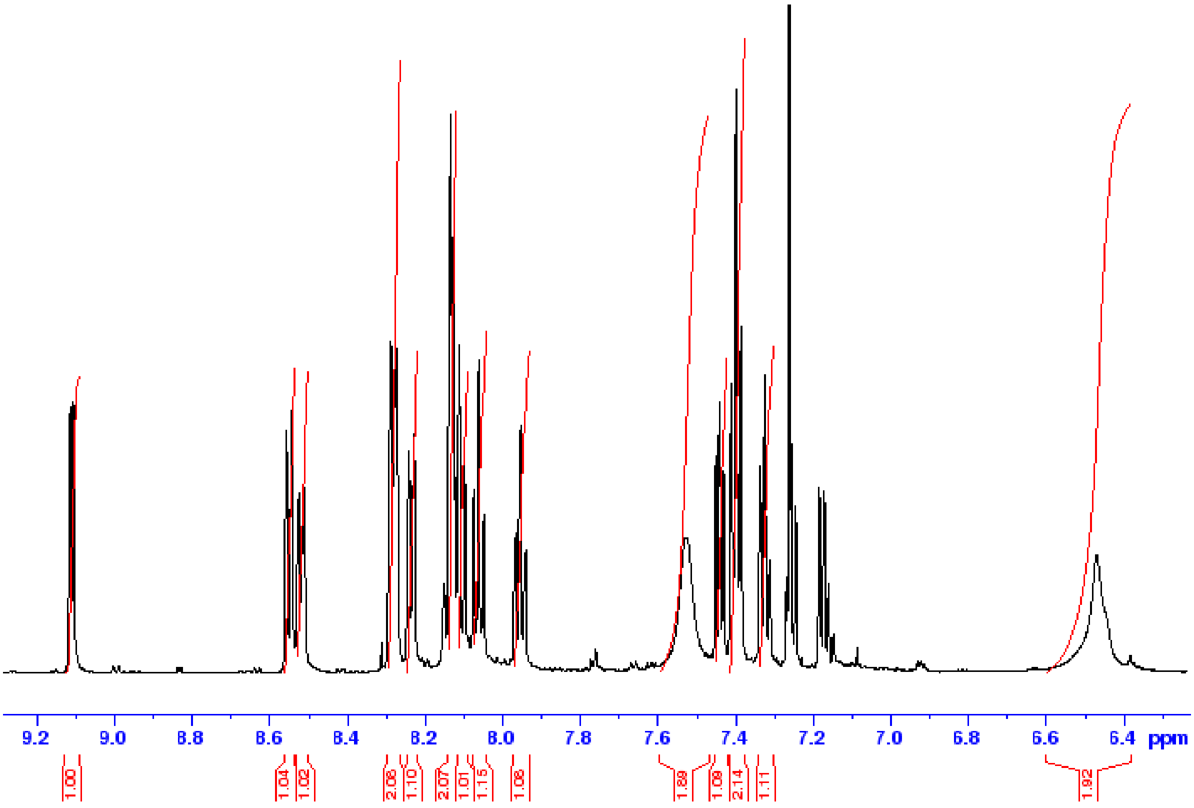

Figure S29. ^{1}H NMR spectrum of Re-NHC-3 in $CDCl_3$, 600 MHz.

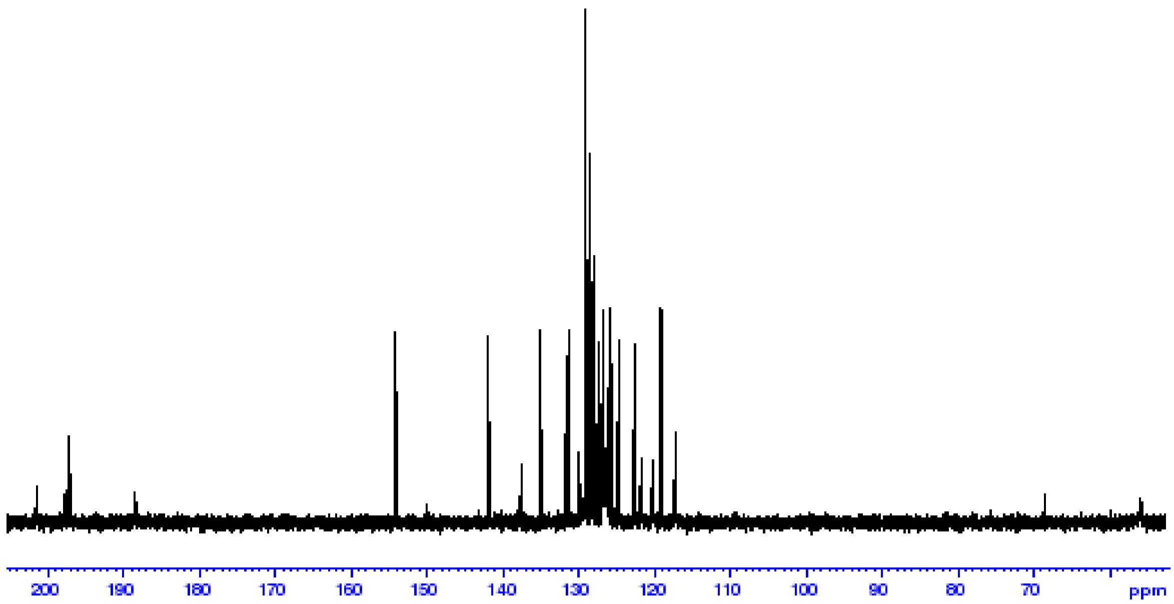

Figure S30. ^{13}C NMR spectrum of Re-NHC-3 in DMSO-d_6, 150 MHz.
